# Delayed Rifampin Administration in the Antibiotic Treatment of Periprosthetic Joint Infections Significantly Reduces the Emergence of Rifampin Resistance

**DOI:** 10.3390/antibiotics10091139

**Published:** 2021-09-21

**Authors:** Ali Darwich, Franz-Joseph Dally, Mohamad Bdeir, Katharina Kehr, Thomas Miethke, Svetlana Hetjens, Sascha Gravius, Elio Assaf, Elisabeth Mohs

**Affiliations:** 1 Department of Orthopaedic and Trauma Surgery, University Medical Centre Mannheim, Medical Faculty Mannheim, University of Heidelberg, Theodor-Kutzer-Ufer 1-3, 68167 Mannheim, Germany; franz.dally@umm.de (F.-J.D.); mohamad.bdeir@umm.de (M.B.); sascha.gravius@umm.de (S.G.); elio.assaf@umm.de (E.A.); elisabeth.mohs@umm.de (E.M.); 2 Institute of Medical Microbiology and Hygiene, University Medical Centre Mannheim, Medical Faculty Mannheim, University of Heidelberg, Theodor-Kutzer-Ufer 1-3, 68167 Mannheim, Germany; katharina.kehr@umm.de (K.K.); thomas.miethke@medma.uni-heidelberg.de (T.M.); 3 Institute of Medical Statistics and Biomathematics, University Medical Centre Mannheim, Medical Faculty Mannheim, University of Heidelberg, Theodor-Kutzer-Ufer 1-3, 68167 Mannheim, Germany; svetlana.hetjens@medma.uni-heidelberg.de

**Keywords:** rifampin, resistance, periprosthetic joint infection, PJI, antibiotic, outcome

## Abstract

Rifampin is one of the most important biofilm-active antibiotics in the treatment of periprosthetic joint infection (PJI), and antibiotic regimens not involving rifampin were shown to have higher failure rates. Therefore, an emerging rifampin resistance can have a devastating effect on the outcome of PJI. The aim of this study was to compare the incidence of rifampin resistance between two groups of patients with a PJI treated with antibiotic regimens involving either immediate or delayed additional rifampin administration and to evaluate the effect of this resistance on the outcome. In this retrospective analysis of routinely collected data, all patients who presented with an acute/chronic PJI between 2018 and 2020 were recorded in the context of a single-center comparative cohort study. Two groups were formed: Group 1 included 25 patients with a PJI presenting in 2018–2019. These patients received additional rifampin only after pathogen detection in the intraoperative specimens. Group 2 included 37 patients presenting in 2019–2020. These patients were treated directly postoperatively with an empiric antibiotic therapy including rifampin. In all, 62 patients (32 females) with a mean age of 68 years and 322 operations were included. We found a rifampin-resistant organism in 16% of cases. Rifampin resistance increased significantly from 12% in Group 1 to 19% in Group 2 (*p* < 0.05). The treatment failure rate was 16% in Group 1 and 16.2% in Group 2 (*p =* 0.83). The most commonly isolated rifampin-resistant pathogen was *Staphylococcus epidermidis* (86%) (*p* < 0.05). The present study shows a significant association between the immediate start of rifampin after surgical revision in the treatment of PJI and the emergence of rifampin resistance, however with no significant effect on outcome.

## 1. Introduction

Periprosthetic joint infections (PJI) are some of the most dreaded complications after total joint arthroplasty (TJA) [1] and are associated with multiple revision operations and a one-year mortality ranging from 8 to 26% [2]. In the current literature, infection rates after primary TJA range from 0.2 to 1.5% [3] and increase after revision TJA to about 5% and up to 15% in cases of mega implants [2,3].

Therapeutic algorithms of PJI include intravenous antibiotics and an operative treatment that differs according to the type of infection (acute or chronic): debridement, antibiotics, and implant retention (DAIR) in cases of acute PJI and implant removal with or without the implantation of an antibiotics-covered spacer in a two/multiple stage exchange regime in cases of chronic PJI [4,5].

Biofilm-active antibiotics are associated with better outcomes regarding infection resolution and joint function [6], and rifampin-containing antibiotic therapy regimes are recommended for PJI caused by Gram-positive microorganisms [7]. Since most PJIs are caused by Gram-positive microorganisms [8] and because of its biofilm activity, rifampin is considered one of the most important antibiotics in the treatment of PJI [4], and antibiotic treatments not involving rifampin were shown to have higher failure rates [9,10,11]. 

In the current literature, both the necessity of long antibiotic treatment intervals and the resulting emergence of antibiotic resistance are well documented. For that reason, one of the most cumbersome complications is the emergence of antibiotic resistance, especially to antibiotic classes commonly used in PJI such as the highly biofilm-active rifampin [4]. In this context, only a few studies have evaluated the risk factors for the emergence of rifampin resistance throughout the treatment of PJI. In particular, the timing of rifampin administration might influence the development of resistance [12].

Many authors recommended administration of rifampin directly after surgical revision in DAIR regimes and after replantation in a two/multiple stage exchange, as they suggest rifampin in these first days might be most effective in preventing biofilm formation on the surface of the prosthesis [5,13,14]. Others prefer to wait until the wounds are dry and the drains are removed [5], or even until the antimicrobial susceptibility of the causing microorganism to rifampin is known and confirmed [15].

The aim of this retrospective clinical study is to compare the incidence of rifampin resistance between two groups of patients with a PJI treated with antibiotic regimens involving either immediate or delayed additional rifampin administration and to evaluate the effect of this resistance on the outcome.

## 2. Results

### 2.1. Study Population

A total of 62 patients (32 females and 30 males) with a mean age of 68.2 ± 11.5 years were included. The first group receiving additional rifampin only after pathogen detection consisted of 25 patients, and the second group treated directly postoperatively with an empiric antibiotic therapy including rifampin consisted of 37 patients.

The recorded parameters of the patients of each of the 2 groups did not statistically differ. 

In total, at a mean follow up of 14.1 ± 11.4 months (6–48 months) (16 ± 13.8 months in Group 1 and 12.6 ± 9.1 months in Group 2), 10 cases (16.1%) of treatment failure (16.1% in Group 1 and 16.2% in Group 2, *p* = 0.83) and 6 deaths (9.7%) were observed. Concerning treatment options, 32% (8/25) of patients in Group 1 and 32.4% (12/37) of Group 2 underwent a switch of treatment strategy from DAIR to prosthesis exchange. One-stage exchange protocol was not performed.

An overview of all the parameters in total and those of each group are to be found in Table 1.

Considering all performed revisions, the mean time period between 2 revisions was 21 ± 22 days. In Group 1, the mean duration from the first surgical revision until detection of the microorganism and release of the definitive antibiogram equated with the first administration of rifampin and lasted 8.3 ± 2.5 days (4–11 days).

The use of intravenous rifampin was higher in Group 2 (65% versus 36%, *p* < 0.05) due to the fact that in this group the rifampin treatment was started empirically. Regarding the subsequent use of oral rifampin, there were no significant differences between groups (*p =* 0.15).

### 2.2. Rifampin Resistance and Subgroup Analysis

In 52 (16%) of the 322 performed operations, a rifampin-resistant organism was found. The prevalence of rifampin resistance varied significantly between the two analyzed groups, where an increase from 12% (14/117 operations) in the first group to 19% (38/205 operations) in the second group was observed (*p* < 0.05). In Group 1, the addition of rifampin took place after the detection of the causing microorganism. The 14 cases with rifampin resistance in Group 1 were cases in which the rifampin resistance developed during the course of the PJI and was only detected later and not at the first revision. A significant correlation with type of infection (acute or chronic) or surgical treatment regime (DAIR or two/multiple stage exchange) was not observed. Only the time of administration of rifampin varied between the two groups. All other covariates did not statistically differ between groups.

The most commonly isolated rifampin-resistant pathogen in both groups was *Staphylococcus epidermidis* with 45 cases (86%) (*p* < 0.05). An overview of all identified rifampin-resistant pathogens can be found in Table 2.

The most administered partner-antibiotics with rifampin in both Groups were vancomycin and flucloxacillin (see Supplementary Materials Table S1). A detailed overview of all detected pathogens per group with their minimum inhibitory concentrations (MIC) can be found in Supplementary Materials Table S2.

The comparison of the baseline characteristics between the patients with a rifampin resistance and those without rifampin resistance in each of the two groups did not show significant differences (Table 3). Further analysis showed that the patients with a rifampin resistance in both Group 1 and Group 2 underwent a higher number of surgical revisions in comparison to the remaining patients in each group (Group 1: 8.6 ± 7.1 vs. 3 ± 2.1 revisions *p* < 0.05 and Group 2: 6.4 ± 4.2 vs. 4.4 ± 4.5 revisions *p* < 0.05). In Group 1, the patients with a rifampin resistance received longer periods of antibiotic treatment (296.1 ± 306 days vs. 91.7 ± 128 days, *p* < 0.05). And in Group 2, compared to the rest of the group, the proportion of chronic PJI was higher in patients with a rifampin resistance (80% vs. 33.3%, *p* < 0.05).

Regarding the outcome, of the 10 cases of treatment failure reported, 4 cases were in Group 1 (16%) (1 case with rifampin resistance, *p* = 0.55) and 6 in Group 2 (16.2%) (4 cases with rifampin resistance, *p* = 0.67). Concerning mortality, of the six death cases observed, three were in Group 1 (one represented a PJI-related death) and three were in Group 2 (one represented a PJI-related death). Causes of PJI-unrelated death included two cases of pneumonia with respiratory failure, one case of heart failure with decompensation and one case of bowel ischemia with septic shock. 

In both groups, the emergence of the rifampin resistance did not seem to significantly affect the outcome (*p* = 0.55 and *p* = 0.67, respectively).

## 3. Discussion

Because of its biofilm activity, rifampin is considered one of the most important antibiotics in the treatment of PJI [4], and antibiotic treatments not involving rifampin were shown to have higher failure rates [9,10,11].

A recent multi-center randomized controlled trial study conducted in Norway in 2020 [16] questioned the efficacy of rifampin in acute staphylococcal PJI treated with DAIR. Karlsen et al. [16] examined the two-year outcome of 48 patients with an acute staphylococcal knee or hip PJI treated with DAIR after being randomized according to the antibiotic treatment with or without additional rifampin therapy. The study could not significantly prove the advantage of rifampin addition. However, the results were skeptically viewed due to the underpowering of the study caused by small sample size. 

A more recent multi-center study published by Beldman et al. [17] in 2021 investigated the outcome of a large cohort of 669 patients with acute staphylococcal PJI and found a significant advantage of antibiotic treatment involving rifampin. In their study, the treatment failure rates increased from 32.2% (131/407 cases) with additional rifampin to 54.2% (142/262) without additional rifampin. 

Both of these recent studies focused on the outcome but did not collect data regarding microbiological failure and did not consider the development of resistance as a confounding factor. Actually, there is paucity of data investigating the timing of rifampin administration and its effect on resistance emergence.

According to a Spanish survey conducted between 1999 and 2008, rifampin resistance was detected in only 0.26% of the methicillin-sensitive *Staphylococcus aureus* (MSSA) and 3.26% of the methicillin-resistant *Staphylococcus aureus* (MRSA) [18]. However, several studies show that rifampin resistance can quickly emerge due to a single-step point mutation caused when rifampin is not adequately used [18,19,20,21,22]. Thus, it is crucial to control adequate use and timing of administration.

The results of the present single-center comparative cohort study retrospectively analyzing routinely collected data show a significant association between the immediate start of rifampin after surgical revision in the treatment of PJI and the emergence of rifampin resistance compared to delayed administration of rifampin. 

Beldman et al. [17] reported an association between immediate start of rifampin therapy and treatment failure. However, the authors could not evaluate the causality of developing resistances on this association since no microbiological data had been collected on the development of pathogen resistance. Our results significantly confirm the association between immediate start of rifampin after surgical revision and emergence of rifampin resistance, which may explain the effect on treatment failure rates seen in the study of Beldman et al. [17].

Although an association between immediate start of rifampin and emergence of rifampin resistance has been shown, a significant effect on the outcome could not be established. A possible explanation may be the fact that the patients in Group 2, where the rates of rifampin resistance were higher, received in many cases highly biofilm-active i.v. antibiotics instead of rifampin compared to patients with a rifampin resistance in Group 1. As mentioned earlier, the most administered partner-antibiotics with rifampin in both groups were vancomycin and flucloxacillin. However, in Group 2 daptomycin was occasionally used [23], which may have had a positive effect on the infect resolution in rifampin-resistant cases. The optimized efficacy of rifampin adjunction to daptomycin in resistant PJI cases has been previously reported [24,25]. 

In the current study, daptomycin was used in 5 patients, all with a PJI caused by rifampin-resistant *S. epidermidis*. However, in comparison to vancomycin (most frequent antibiotic partner), the revision rates (*p* = 0.43) and failure rates (*p* = 1.00) did not show any significant differences. The limited significance is due to the small number of cases in this subgroup.

In the current study, the further analysis of the patients with rifampin resistance in each group showed that those with rifampin resistance underwent significantly more revisions than their counterparts without rifampin resistance in each group (Group 1: 8.6 ± 7.1 vs 3 ± 2.1 revisions *p* < 0.05 and Group 2: 6.4 ± 4.2 vs 4.4 ± 4.5 revisions *p* < 0.05). These results are in line with those published by Achermann et al. [12], where an association was observed between ≥3 surgical revisions and the emergence of rifampin resistance (*p* < 0.05) in staphylococcal PJI. Achermann et al. [12] suggest an inoculation of rifampin-resistant strains from the skin to the joint as a possible route of infection. These results associate the increased number of surgeries with the emergence of rifampin resistance; however, the hypothesis that the delayed rifampin administration leads to an increased number of revisions is not supported (4.8 ± 4.6 revisions in Group 1 vs 5.6 ± 4.4 revisions in Group 2, *p* = 0.10). This means that although the timing of rifampin administration affects the emergence of rifampin resistance, the increased number of revisions may have been the result of insufficient debridement surgeries and a deficient initial “source control” independent from the rifampin therapy. In other words, even if the rifampin administration was delayed, a suboptimal debridement could still lead to more necessary revisions and ultimately may play a role in the emergence of rifampin resistance.

One of the limitations of this study is the low level of evidence due to its retrospective design. Another limitation is the rather small number of patients per study group, which limited the statistical significance of the analysis of some associations, especially when the rifampin-resistant pathogens were subcategorized according to the species involved.

The role of rifampin in the management of PJI, especially that caused by *Staphylococcus aureus* and treated by DAIR, is well documented in the literature [26,27]. In the current study, patients with a PJI caused by *Staphylococcus aureus* and managed with DAIR in each of the groups were separately analyzed. In total, 6 cases of *Staphylococcus aureus* in Group 1 and 9 cases in Group 2 were observed, of which 3/6 (50%) and 6/9 (66.7%) were managed with DAIR (*p* = 0.62). One case of treatment failure in each of the 2 subgroups was recorded (*p* = 1.00), and none of the PJIs caused by *Staphylococcus aureus* developed a rifampin resistance. The observed results after DAIR and combination antibiotic therapy in combination with rifampin is comparable with data in the literature [26,27]; however, as mentioned earlier, because of the small number of patients per study subgroup the results were not significant. 

The absence of emerging rifampin resistance in PJI cases caused by *Staphylococcus aureus* may be due to their low total incidence in the included patients. In the present study, an incidence of 24% was observed, which is lower than that reported by Pulido et al. [3], where an incidence of 38% was documented (19% methicillin-sensitive *Staphylococcus aureus* and 19% methicillin-resistant *Staphylococcus aureus*). The low incidence of *Staphylococcus aureus* PJI was investigated by Ribau et al. [28] in a recent meta-analysis involving 32 publications. The study concluded that preoperative screening and decolonization measurements reduce the risk of *Staphylococcus aureus* infection. This may explain the lower incidence in the patients of the current study since these measurements, including preoperative nasal and whole-body decolonization as well as preoperative screening, form part of the standard of care and preoperative preparation in our hospital.

The patients included in this study were not randomized and the inclusion was performed in near but different time frames. This time difference may have had an effect on the nature of the infecting organisms and their resistance profiles, which is considered a third limitation of the study. Another limitation is the heterogenous measurement criteria used to assess patient outcomes in similar studies investigating PJI [29,30,31]. This lack of homogeneity may have limited the comparability of the results of the current study with those in the literature. A last limitation lies in the fact that different surgeons were involved in the surgical treatment of the included patients. Even when the choice of the most appropriate treatment option follows a previously well-defined internal algorithm, some technical decisions were made at the discretion of the treating surgeon, which may have had a small effect on the course of the infection.

## 4. Materials and Methods

### 4.1. Study Population and Antibiotics

In this retrospective analysis of routinely collected data, all patients who presented with an acute/chronic PJI between 2018 and 2020 were recorded in the context of a single-center comparative cohort study. There were no exclusion criteria. This study has been reported in line with the STROBE Guidelines [32].

The diagnostic criteria for a PJI were defined according to the guidelines of the Muscoloskeletal Infection Society (MSIS) [33].

The patients were categorized in 2 groups; the first group included all patients with a PJI presenting from 01/2018 to 06/2019, and the second group included all patients with a PJI presenting from 07/2019 to 12/2020.

The patients of each of the two groups were given a different regime of antibiotic therapy. The inclusion to each group and the selection of the used antibiotic regime was not randomized. The inclusion in each of the groups was done according to the time of presentation to the hospital. The first group of patients received an empiric therapy without rifampin directly after the surgical revision and rifampin was added only after pathogen detection in the microbiological examination of the specimens collected intraoperatively, while the second group was treated directly postoperatively with an empiric antibiotic therapy including rifampin.

Patients with a PJI caused by a rifampin-resistant strain detected directly in the first revision were excluded, since the rifampin resistance in these cases was independent of the timing of rifampin administration. 

Patients with a PJI caused exclusively (in the whole course of the infection) by microorganisms where rifampin is not effective such as Gram-negative bacilli, were also excluded.

According to the internal hospital protocol, rifampin was administered in a dose of 300 mg twice daily during the total duration of the antibiotic treatment (2 weeks intravenous and 4 weeks orally).

None of the patients presented with an allergy or intolerance preventing the use of rifampin, and none of the patients developed a hepatotoxicity or organ toxicity leading to an unplanned early discontinuation of the rifampin treatment.

The values presented in this study are based on the number of detected pathogens rather than the number of patients. Some patients presented with a polymicrobial infection while others presented with different pathogens in each operative revision; therefore, the number of cases was defined by the number of pathogens to provide a more precise analysis.

### 4.2. Recorded Parameters

The recorded parameters included age, sex, operated side, involved joint, body-mass-index (BMI), preoperative comorbidity using ASA (American society of Anesthesiologists) Physical Status Classification System [34], type of infection (acute or chronic), operative and antibiotic therapy regime and the time period of intravenous and oral antibiotic therapy. 

The detected pathogens were also documented. Rifampin resistance was determined according to the guidelines of the Clinical & Laboratory Standards Institute (CLSI) using the Vitek II system (bioMerieux, Nürtingen, Germany) and documented in microbiology laboratory reports. Rifampin resistance of the same pathogen in the same patient was considered one case, even if it was detected several times in one or more revisions in the course of the infection. The time of rifampin administration in relation to the operative revision and in relation to the time of microorganism detection as well as the duration of antibiotic therapy involving rifampin have been registered.

Based on the guidelines of the Infectious Diseases Society of America, acute infections were defined as those appearing within 4 weeks after primary implantation or causing symptoms of less than 3 weeks. Chronic infections were the remaining infections occurring beyond these time limits [35]. Labeling of the infections as acute or chronic was done according to these criteria directly on the first presentation, since at this stage the classification of the infection plays an essential role in the choice of the appropriate surgical strategy. Therefore, the results shown in Table 1 and Table 3 refer to this first labeling. However, a transition from acute to chronic, such as in the case of unsatisfactory results after the first surgical intervention and the need of a second revision, is clearly possible.

Therapy regimes were based on the guidelines of the International Consensus Group on Periprosthetic Joint Infection [36] and included debridement, antibiotics, and prosthesis retention (DAIR) in cases of acute PJI or exchange of prosthesis in the context of a two/multiple stage exchange regime with or without cement spacer implantation in cases of chronic PJI. One-stage exchange protocol was not performed.

A minimum of 4 pairs of deep tissue specimens were obtained intraoperatively. Each pair was obtained from the same anatomical site. The specimens of each pair were divided and sent either for histopathological analysis or for microbiological culturing to allow result matching. 

Cultures with no pathogen growth after an extended incubation period of minimum 10 days were considered negative [37]. Signs of PJI in the histopathological examination of the specimens were defined according to the classification of Morawietz et al. [38]. The results and the detected pathogens were registered. 

Patient outcomes were categorized based on the classification proposed by Parvizi et al. [29] and were defined as:

- Infect resolution: no clinical signs of infection, CRP < 10 mg/L;
- Treatment failure: persistent clinical signs of infection after the definitive revision of the PJI, infection recurrence caused by the same or different pathogen or need of a subsequent surgery owing to infection after the definitive revision of the PJI, chronic antibiotic suppression, death due to a PJI-related sepsis.

Cases requiring a change of treatment strategy such as a switch from DAIR to prosthesis exchange strategy, for example, were not defined as treatment failure as long as they meet the criteria mentioned above in the entire follow-up period after the definitive revision of the PJI.

The reported number of revisions was defined as the total number of performed revisions since the first presentation with the PJI.

The included patients were evaluated at fixed times postoperatively (6 weeks, 3 months, 6 months, 1 year, 2 years and then every 2 years). The results reported in the presenting study are the ones recorded in the last follow-up examination.

### 4.3. Statistical Analysis

All statistical calculations were performed using SAS software, release 9.4 (SAS Institute Inc., Cary, NC, USA). Quantitative data are presented as the mean and standard deviation; discrete variables, the median and range are given. For approximately normally distributed data, two sample t-tests were used in order to compare the mean values of two groups. For skewed variables Mann–Whitney U-tests were performed instead. In order to compare groups regarding qualitative parameters, a Chi-square test or Fisher’s exact test was used. Correlation analyses were determined by the Spearman correlation coefficient. Statistical significance has been assumed for *p* values less than 0.05.

### 4.4. Ethics Approval

This study was approved by the Ethics Committee of clinical research at our institution (Ethics Committee II, University Medical Centre Mannheim, Medical Faculty Mannheim, Heidelberg University, Theodor-Kutzer-Ufer 1–3, 68167, Mannheim, Approval 2021-814) and performed in accordance with the local ethical standards and the principles of the 1964 Helsinki Declaration and its later amendments.

## 5. Conclusions

The results of the present retrospective single-center comparative cohort study show a significant association between the immediate start of rifampin after surgical revision in the treatment of PJI and the emergence of rifampin resistance compared to delayed administration of rifampin, however with no significant effect on outcome. An additional independent factor is the significantly higher number of surgical revisions in the groups of patients with emerging rifampin resistance.

## Figures and Tables

**Table 1:** Patient data.

Parameter		Total	Group 1	Group 2	*p*-Value *
Number of patients	n (%)	62	25 (40.3%)	37 (59.7%)	--
Number of revisions	n (%)	322 (100%)	117 (36%)	205 (64%)	0.30
Age	Mean ± SD years	68.2 ± 11.5	68.4 ± 2.8	68.1 ± 9.7	0.64
Sex	Males n (%)	30 (48%)	12 (48%)	18 (49%)	1.00
	Females n (%)	32 (52%)	13 (52%)	19 (51%)	
Side	Right n (%)	31(50%)	11 (44%)	20 (54%)	0.61
	Left n (%)	31 (50%)	14 (56%)	17(46%)	
Involved joint	Hip n (%)	39 (63%)	19 (76%)	20 (54%)	0.07
	Knee n (%)	23 (37%)	6 (24%)	17 (46%)	
BMI	Mean ± SD Kg/m^2^	30.2 ± 8.9	28.9 ± 1.6	31.1 ± 9.5	0.33
ASA Score	ASA Score 1 n (%)	1 (2%)	1 (2%)	0 (0%)	0.74
	ASA Score 2 n (%)	25 (40%)	9 (36%)	16 (43%)	
	ASA Score 3 n (%)	33 (53%)	14 (60%)	19 (52%)	
	ASA Score 4 n (%)	3 (5%)	1 (2%)	2 (5%)	
Type of infection	Acute n (%)	33 (53%)	15 (60%)	18 (49%)	0.61
	Chronic n (%)	29 (47%)	10 (40%)	19 (51%)	
Number of revisions per patient	Mean ± SD	5.3 ± 4.4	4.8 ± 4.6	5.6 ± 4.4	0.10
Operative therapy	DAIR n (%)	25/62 (40.3%)	10/25 (40%)	15/37 (40.5%)	0.17
	Prosthesis exchange n (%)	17/62 (27.4%)	7/25 (28%)	10/37 (27%)	
	Therapy switch n (%)	20/62 (32.3%)	8/25 (32%)	12/37 (32.5%)	
Number of operations with detected rifampin resistance	n (%)	52/322 (16%)	14/117 (12%)	38/205 (19%)	<0.05
Interval between surgical revision and rifampin administration	Mean ± SD days	--	8.3 ± 2.5	0	*--*
Treatment failure	n (%)	10/62 (16.1%)	4/25 (16%)	6/37 (16.2%)	0.83

SD—Standard Deviation, BMI—Body Mass index, ASA—American Society of Anesthesiology, DAIR—debridement, antibiotics, implant retention, * *p*-value < 0.05 was considered as statistically significant.

**Table 2:** Identified rifampin-resistant pathogens.

Rifampin-Resistant Pathogen	Group 1	Group 2	*p*-Value *
*S. epidermidis*	11 (21%)	34 (65%)	<0.05
*S. hominis*	1 (2%)	1 (2%)	0.51
*S. heamolyticus*	2 (4%)	2 (4%)	0.32
*S. capitis*	0 (0%)	1 (2%)	1.00

S.—*Staphylococcus*, * *p*-Value < 0.05 was considered statistically significant.

**Table 3:** Subgroup data.

Parameter		Group 1		*p*-Value *	Group 2		*p*-Value *
		No rifampin Resistance	Rifampin Resistance		No rifampin Resistance	Rifampin Resistance	
Number of pathogens **	n (%)	103 in 117 revisions (88%)	14 in 117 revisions (12%)	*--*	157 in 205 revisions (81%)	38 in 205 revisions (19%)	*--*
Number of patients		16	9		22	15	
Age	Mean ± SD years	67.5 ± 15.1	70.6 ± 8.9	0.40	66.4 ± 10.2	69.1 ± 9.5	0.63
Sex	Males n (%)	8/16 (50%)	4/9 (44.4%)	0.66	12/22 (54.5%)	6/15 (40%)	0.51
	Females n (%)	8/16 (50%)	5/9 (55.6%)		10/22 (45.5%)	9/15 (60%)	
Side	Right n (%)	8/16 (50%))	3/9 (33.3%)	0.66	12/22 (54.5%)	9/15 (60%)	1.00
	Left n (%)	8/16 (50%))	6/9 (66.7%)		10/22 (45.5%)	6/15 (40%)	
Involved joint	Hip n (%)	12/16 (%)	7/9 (77.8%)	0.71	11 (50%)	11/15 (73.3%)	0.29
	Knee n (%)	4/16 (%)	2/9 (22.2%)		11 (50%)	4/15 (26.7%)	
BMI	Mean ± SD Kg/m^2^	29.4 ± 8.8	27.9 ± 6.4	0.92	34.1 ± 11	29.5 ± 8	0.29
ASA Score	Mean ± SD	2.7 ± 0.6	2.7 ± 0.5	0.75	2.6 ± 0.7	2.7 ± 0.6	0.65
Anticoagulation at admission	Yes n (%)	6/16 (%)	3/9 (33.3%)	0.66	6/22 (27.3%)	8/15 (53.3%)	0.17
	No n (%)	10/16 (%)	6/9 (66.7%)		16/22 (72.7%)	7/15 (46.7%)	
Treatment before admission	Yes n (%)	5/16 (%)	1/9 (11.1%)	1.00	6/22 (27.3%)	3/15 (20%)	0.71
	No n (%)	11/16 (%)	8/9 (88.9%)		16/22 (72.7%)	12/15 (80%)	
Type of infection	Acute n (%)	11/16 (%)	4/9 (44.4%)	0.38	15 (66.7%)	3 (20%)	<0.05
	Chronic n (%)	5/16 (%)	5/9 (55.6%)		7 (33.3%)	12 (80%)	
Number of operations per patient	Mean ± SD	3 ± 2.1	8.6 ± 7.1	<0.05	4.4 ± 4.5	6.4 ± 4.2	<0.05
Duration antibiotic treatment	Mean ± SD days	91.7 ± 127.9	296.1 ± 306.3	<0.05	229.6 ± 472.8	300.1 ± 357.2	0.13
Treatment failure	n (%)	3/16 (18.8%)	1/9 (11.1%)	0.55	2/22 (9.1%)	4/15 (26.7%)	0.67

SD—Standard Deviation, BMI—Body Mass index, ASA—American Society of Anesthesiology, DAIR—debridement, antibiotics, implant retention, * *p*-value < 0.05 was considered as statistically significant. ** Rifampin resistance of the same pathogen in the same patient was considered as one case, even if it was detected several times in one or more revisions in the course of the infection. The numbers stated here refer to the sum of all resistant staphylococci, and each species was counted once per patient.

## Data Availability

The data presented in this study are available on request from the corresponding author.

## Supplementary Materials

The following are available online at https://www.mdpi.com/article/10.3390/antibiotics10091139/s1, Table S1: Number of patients per group receiving each antibiotic partner according to route of administration, Table S2: Number of patients per group and per detected pathogens.
