# Supplementary material for: Delayed Rifampin Administration in the Antibiotic Treatment of Periprosthetic Joint Infections Significantly Reduces the Emergence of Rifampin Resistance
Source: Antibiotics (Basel). 2021 Sep 21;10(9):1139. doi: 10.3390/antibiotics10091139 (PMC8469909; doi:10.3390/antibiotics10091139)
Supplement: Supplementary file 1 [file antibiotics-10-01139-s001.zip › antibiotics-1362193-supplementary.pdf]

Supplementary Materials

Table S1. Number of patients per group receiving each antibiotic partner according to route of administration.

	Group 1	Group 2
**Intravenous**		
Vancomycin	15	28
Flucloxacillin	9	8
Clindamycin	7	3
Ceftriaxon	4	6
Sultamicillin	2	4
Ampicillin	2	0
Meropenem	2	7
Fucidic acid	2	2
Penicillin	1	1
Cefuroxim	1	0
Ciprofloxacin	1	1
Levofloxacin	1	1
Fosfomycin	1	1
Amoxicillin	1	0
Piperacillin/Tazobactam	1	0
Teicoplanin	0	4
Daptomycin	0	5
Cephazolin	0	1
Cefotaxim	0	1
Ceftazidim	0	1
**Oral**		
Linezolid	13	28
Clindamycin	6	4
Amoxicillin/Clavulanate	5	0
Penicillin	3	0
Cefuroxim	2	2
Fucidic acid	2	2
Fosfomycin	1	1
Cefotaxim	1	0
Ciprofloxacin	0	5
Doxycyclin	0	1
Flucloxacillin	0	4

Table S2. Number of patients per group and per detected pathogens.

	Group 1	Rifampin MIC (µg/ml)	Group 2	Rifampin MIC (µg/ml)
Staphylococcus epidermidis	10	≤ 0.5	19	≤ 0.5
Enterococcus faecalis	6	≤ 0.5	7	≤ 0.5
Staphylococcus aureus	6	≤ 0.5	9	≤ 0.5
Staphylococcus haemolyticus	5	≤ 0.5	7	≤ 0.5
Staphylococcus capitis	3	≤ 0.5	4	≤ 0.5
Enterococcus faecium	3	≤ 0.5	1	≤ 0.5
Staphylococcus hominis	2	≤ 0.5	2	≤ 0.5
Pseudomonas aeruginosa	2	x	1	x
Proteus mirabilis	2	x	2	x
Staphylococcus pettenkoferi	1	≤ 0.5	1	≤ 0.5
Klebsiella aerogenes	1	x	0	x
Klebsiella pneumoniae	1	x	5	x
Escherichia coli	1	x	2	x
Staphylococcus lugdunensis	1	≤ 0.5	0	x
Streptococcus dysgalactiae	1	x	0	x
Moraxella bovis	1	≤ 0.5	0	x
Staphylococcus cohnii	1	≤ 0.5	1	≤ 0.5
Cutibacterium acnes	1	≤ 0.5	1	≤ 0.5
Enterobacter cloacae	0	x	3	x
Acinetobacter lwoffii	0	x	1	x
Streptococcus agalactiae	0	x	1	x
**Rifampin resistant**				
Staphylococcus epidermidis	10	≥ 32	31	≥ 32
Staphylococcus epidermidis	1	≥ 16	3	≥ 16
Staphylococcus haemolyticus	2	≥ 32	2	≥ 32
Staphylococcus hominis	1	8	1	2
Staphylococcus capitis	0	x	1	≥ 32

* MIC Minimum Inhibitory Concentration
